# The stabilizing effect of shear thinning on the onset of purely elastic instabilities in serpentine microflows†
†Electronic supplementary information (ESI) available. See DOI: 10.1039/c6sm00326e



**DOI:** 10.1039/c6sm00326e

**Published:** 2016-05-17

**Authors:** Laura Casanellas, Manuel A. Alves, Robert J. Poole, Sandra Lerouge, Anke Lindner

**Affiliations:** a PMMH , UMR 7636 CNRS – ESPCI Paris – Université Pierre et Marie Curie Université Paris Diderot , 10 rue Vauquelin , F-75231 Paris CEDEX 05 , France . Email: anke.lindner@espci.fr; b Laboratoire Matière et Systèmes Complexes , CNRS UMR 7057-Université Paris Diderot , 10 rue Alice Domond et Léonie Duquet , 75205 Paris CEDEX 05 , France; c Departamento de Engenharia Química , CEFT , Faculdade de Engenharia da Universidade do Porto , Rua Dr Roberto Frias , 4200-465 Porto , Portugal; d School of Engineering , University of Liverpool , Brownlow Hill , Liverpool L69 3GH , UK

## Abstract

We determine both experimentally and numerically the onset of elastic flow instabilities in viscoelastic polymer solutions with different levels of shear thinning. Previous experiments realized in microfluidic serpentine channels using dilute polymeric solutions showed that the onset of elastic instabilities strongly depends on the channel curvature. The scaling dependence is well captured by the general instability scaling criterion proposed by Pakdel and McKinley [*Phys. Rev. Lett.*, 1996, **76**, 2459:1–4]. We determine here the influence of fluid shear thinning on the onset of such purely-elastic flow instabilities. By testing a set of polyethylene oxide solutions of high molecular weight at different polymer concentrations in microfluidic serpentine channels we observe that shear thinning has a stabilizing effect on the microfluidic flow. Three-dimensional numerical simulations performed using the White–Metzner model predict similar trends, which are not captured by a simple scaling analysis using the Pakdel–McKinley criterion.

## Abstract 2

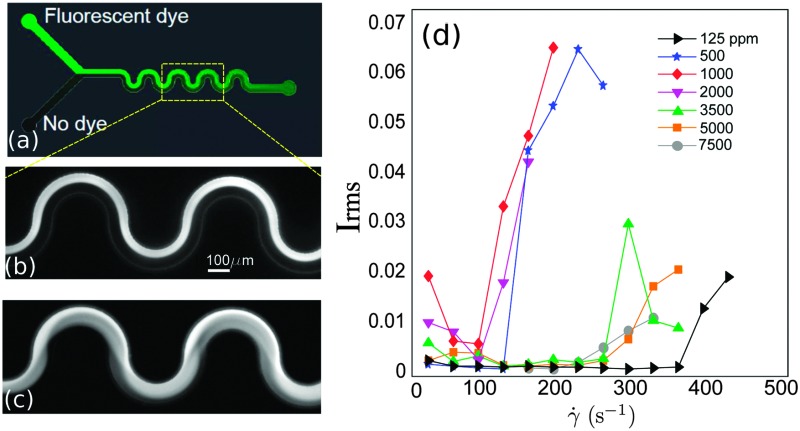
We determine both experimentally and numerically the onset of elastic flow instabilities in viscoelastic polymer solutions with different levels of shear thinning.

## 1 Introduction

It is well known that complex fluid flows may develop non-trivial states beyond a critical flow rate which, in general, depends on the particular fluid and flow geometry in question. When inertial effects are unimportant, as in fully-developed or viscometric flows, in extremely viscous fluids or in microscale flows where the kinetic energy and hence Reynolds number are vanishingly small, such instabilities are termed “purely-elastic”. Given the prevalence of such types of flows for complex fluids, purely-elastic instabilities have been observed in a wide-range of situations. For example, at the microscale, these instabilities have been used to enhance mixing^1,2^ which is otherwise absent in the corresponding flow of an equally viscous Newtonian fluid. In viscometric flows they have been extensively studied (see Shaqfeh^3^ for a comprehensive review), due to the advantage of simplifying the flow kinematics in order to better understand the physical mechanisms at play, but also from a practical standpoint as such instabilities are generally undesirable in viscometric measurements (and usually preclude the determination of material properties above their onset). Such instabilities also occur in industrial applications, *e.g.* polymer extrusion or ink jet printing, and a good theoretical understanding and the ability to control their onset is of wide relevance.

Most previous studies on purely-elastic flow instabilities, in a sensible attempt to separate out effects of elasticity from shear thinning, have used constant-viscosity elastic liquids, so-called Boger fluids.^4–6^ However, the vast majority of viscoelastic fluids, both in nature (*e.g.* blood,^7^ synovial fluid or hyaluronic acid,^8^ snail mucus^9^) and synthetic fluids used in different industries (polymer solutions^10^ or wormlike surfactant solutions used in personal products,^11^ foodstuffs or pharmaceutical and chemical settings^12^), often display strong shear thinning of the shear viscosity, especially at high shear rates. Physically, shear thinning arises as a direct consequence of the fluid microstructure, especially when the fluid can no longer be considered to be dilute (in some sense) and interactions occur: in colloidal suspensions it may arise *via* alignment of suspended particles under flow; in polymeric fluids or wormlike micellar solutions *via* alignment or disentanglement.

Previous studies which have used dilute polymer solutions, *i.e.* essentially Boger fluids, have revealed much about onset conditions, physical mechanisms and scaling of elastic flow instabilities. In particular, it is now commonly accepted that the main mechanism responsible for the appearance of most purely-elastic instabilities is a combination of flow-induced tensile stresses and the streamline curvature (elegantly captured in the Pakdel–McKinley criterion^13^). For example, Zilz *et al.* ^14^ were able to use the Pakdel–McKinley criterion to systematically test the effect of the flow curvature on the appearance of purely-elastic instabilities in microfluidic serpentine (wavy) channels for dilute polymer solutions. Good agreement was observed between the analysis based on simple scaling arguments, microfluidic experiments and three-dimensional numerical calculations using the upper-convected Maxwell (UCM) model. The aim of the current paper is to extend this analysis to incorporate more realistic rheology, specifically shear-thinning effects.

To date, the influence of non-linear rheology, and in particular shear thinning of the shear viscosity, on the onset of purely-elastic flow instabilities has not been studied in detail. McKinley *et al.* ^15^ discussed how shear thinning, solvent viscosity and spectra of relaxation times might be incorporated into the instability criterion considering cone-and-plate flows (*i.e.* where the shear rate and the radius of curvature are known). We will show here that the correction suggested to incorporate shear-thinning effects only works for small amounts of shear thinning and fails at higher levels. Other studies which have probed shear-thinning effects include the works of Larson *et al.* ^16^ who undertook a combined linear stability analysis with the K-BKZ model and experimental measurements in the Taylor–Couette geometry. They found shear thinning tended to increase the critical Deborah number, defined as the product between the shear rate and the longest relaxation time, while decreasing the critical value of the ratio between the first normal-stress difference (*N*_1_) and the shear stress. The experiments exhibited instabilities that occur at shear rates 20–45% lower than those predicted by the axisymmetric linear stability analysis but this discrepancy was attributed to the appearance of non-axisymmetric modes. The effect of polymer concentration, which incorporates changes in shear thinning once out of the dilute regime, on the onset of elastic turbulence in Taylor–Couette flow was studied by Jun and Steinberg.^17^ More recently, Nicolas and Fuchs^18^ predicted, using the White–Metzner model, a stabilizing effect on the onset of elastic flow instabilities in dense colloidal suspensions, which was attributed to the significant shear-thinning behaviour of such systems. Theoretical investigations performed by Wilson and Rallison^19^ using also the White–Metzner model showed that for strongly-shear-thinning fluids (with a power-law exponent, *n* < 0.3) the flow could become elastically unstable in a straight channel (in the absence of a curvature), due to the large degree of shear thinning of the fluid. These predictions were supported by experimental work done by Bodiguel *et al.* ^20^ using polymer solutions.

As touched on briefly above, in this work we aim to assess the influence of shear thinning (0.6 < *n* < 1) on the onset of a purely-elastic flow instability in microfluidic serpentine channels. The use of microfluidic channels enables us to use water-based fluids, which are inherently cheap, safe and easily disposed of, whilst still keeping inertial effects small. In particular, polymeric solutions are of great interest since they have been extensively characterized in the literature.^21–30^ We are able to systematically modify the degree of shear thinning by tuning the polymer concentration and, in particular, by crossing the critical overlap concentration into the semi-dilute regime. As the foregoing has made clear, our results have many important potential applications – especially for ‘real’ fluids which are invariably shear thinning – as well as identifying a possible limitation of the Pakdel–McKinley scaling criterion which suggests the need for a generalization of the theoretical framework to predict the experimental findings observed here.

## 2 Materials and methods

### 2.1 Polymer solutions

Solutions of polyethylene oxide (PEO, from Sigma Aldrich) with a molecular weight of *M*_W_ = 4 × 10^6^ g mol^–1^ were prepared in a water/glycerol (75% – 25% in weight) solvent. The solvent viscosity at *T* = 23 °C is *η*_s_ = 2.1 mPa s (data not shown). We tested a set of solutions of different polymer concentrations: *c* = 125, 500, 1000, 2000, 3500, 5000, and 7500 ppm (w/w). The overlap concentration of this polymer in water is c* ≃ 550 ppm.^26^

Rheological measurements presented in Section 3 were performed using both a strain-controlled rheometer (ARES-G2, from TA Instruments), with a 50 mm diameter and 2.3° titanium cone geometry, as well as a stress-controlled rheometer (Physica MCR 501, from Anton Paar), with a 60 mm diameter and 1° titanium cone geometry.

### 2.2 Microfluidic flow geometry

**Fig. 1:**
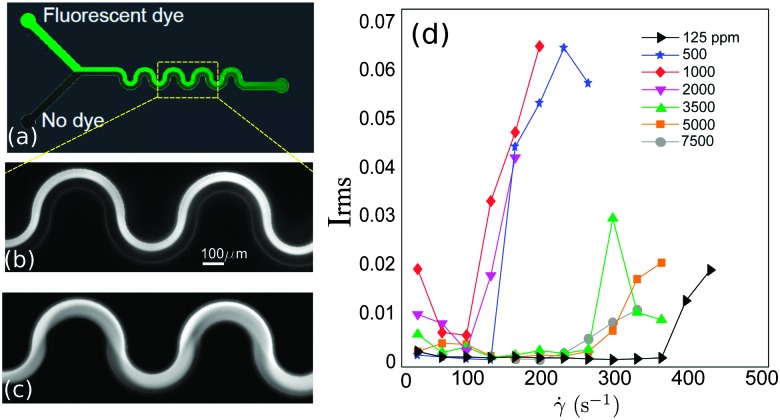
(a) Microfluidic serpentine channel. Illustration of laminar (b) and unstable (c) flows. (d) Rms of the intensity fluctuations measured for increasing applied flow rates and for different polymer concentrations.

We tested the polymer solutions in serpentine microchannels consisting of nine half loops. A picture of the channel geometry is shown in Fig. 1a. The channels have a nearly square cross-section, with a width of *W* = 100 μm, and a height of *H* = 95 ± 3 μm. The average radius of curvature (measured at the centreline of the channel, *R* = *R*_i_ + *W*/2, where *R*_i_ is the inner radius) is *R* = 100 μm. In curved channels, the laminar velocity profile along the channel width displays a slight asymmetry with respect to a Poiseuille flow, with the maximum velocity being shifted towards the inner radius of the loops. Further details of the laminar base flow are provided in Zilz *et al.* ^14^ Note as well that in the serpentine channel the sign of the curvature changes from positive to negative in each half loop. The microchannels were fabricated in polydimethylsiloxane (PDMS), using standard soft-lithography microfabrication methods,^31^ and mounted on a glass slide. The fluid was injected into the channel *via* two inlets using two glass syringes (Hamilton, 500 μl each) that were connected to a high-precision syringe pump (Nemesys, from Cetoni GmbH). The experimental protocol consisted of stepped ramps of the flow rate increasing from 2 μl min^–1^ up to a maximum of 40 μl min^–1^, with a flow rate step of 2 μl min^–1^. The resolution of the imposed flow rate, which was confirmed independently using a flow sensor (SLI-0430 Liquid flow meter, from Sensirion), was controlled at a precision of ±0.5 μl min^–1^. In order to ensure flow steadiness, and to ensure that any possible initial transient was mitigated, the step duration of each flow rate was set to 120 s and image acquisition was performed over the last 60 s of each step.

### 2.3 Flow visualization

The flow was visualized using an inverted fluorescence microscope (Axio Observer A1, from Zeiss) coupled to a CCD monochromatic camera (model PLB741F, from Pixelink) which was used for image acquisition. The frame rate was set to 2 frames per second. The goal of our visualization technique was to discriminate between a laminar (steady) and an unstable (time-dependent) flow. This was achieved by simultaneously injecting the polymeric solution through the two inlets of the microchannel. In one of the inlets the fluid had been previously labeled with a fluorescent dye (fluorescein), so that the labeled and non-labeled streams became easily distinguishable. In the laminar regime the flow is steady and the interface between the labeled and non-labeled solutions is well defined (and becomes only slightly blurred due to diffusion occurring on long time scales). In the unstable flow regime an oscillatory flow pattern is observed and efficient mixing between the labeled and non-labeled solutions occurs. Fig. 1b and c present an example of a steady laminar and an unstable flow regime (illustrative movies can be found in Lindner^32^).

The onset of flow instability is visually determined by the onset of intensity fluctuations in the channel. In order to set precisely the critical imposed flow, *Q*_*c*_, for which the flow becomes time-dependent, we measured the root-mean-square of the intensity fluctuations in time (*I*_rms_)1
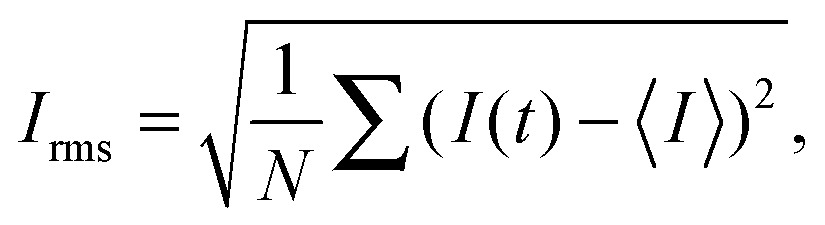
where *N* = 120 is the number of frames analyzed per flow rate step. Fluctuations were measured within a fixed squared region with lateral length *W*/3, located in the last serpentine loop. In Fig. 1d we show *I*_rms_ as a function of the imposed shear rate for different polymer concentrations, for one example. Whereas for the lowest polymer concentrations the onset of the time-dependent flow occurs sharply, for the largest polymer concentrations (*c* ≥ 5000 ppm) the transition from a laminar to a time-dependent flow sets in more gradually. The critical shear rate was determined as *γ̇*_*c*_ = *U*/*W*, where *U* is the mean velocity inside the channel, *U* = *Q*_*c*_/(*WH*).

At the onset of flow instabilities the Reynolds number (Re = *ρUW*/*η*(*γ̇*)), where *ρ* is the fluid density, and *η*(*γ̇*) the rate dependent shear viscosity, is small, ranging from 0.05 (for the highest polymer concentration) up to 1.7 (for the lowest concentration). Therefore, in our microfluidic flow experiments inertial effects can be disregarded.

## 3 Results

### 3.1 Relaxation time for shear-thinning fluids

In order to test the influence of non-linear rheology on flow stability we use polymer solutions in the dilute and semi-dilute regimes. Dilute polymeric solutions (*c* < c*) can be approximately described by the Oldroyd-B model,^33^ with a constant shear viscosity. The viscosity *η* is dominated by the Newtonian solvent contribution, *η*_s_. This model predicts a quadratic increase of the first normal-stress difference (*N*_1_) with shear rate, *N*_1_ = 2(*η* – *η*_s_)*λγ̇* ^2^ (where *λ* is the relaxation time). As the polymer concentration is increased the rheological response of the solutions becomes shear-rate dependent. This non-linear rheological response is no longer well described by the Oldroyd-B model, but can be described using the White–Metzner constitutive equation,^34^ which assumes a shear-rate dependent shear viscosity and relaxation time, here described using Carreau-type models.^10^ The material functions for steady shear flows for a White–Metzner fluid are the following:2
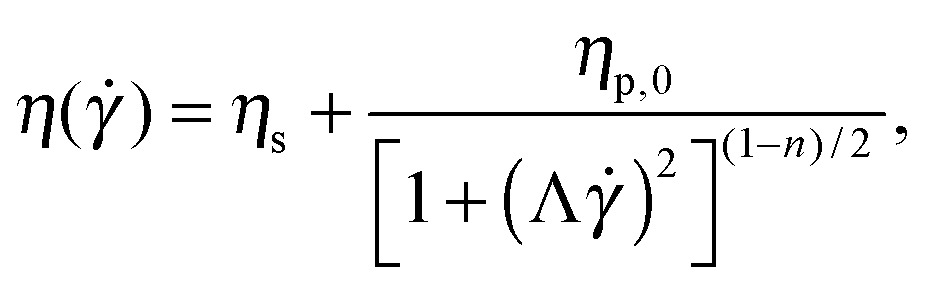

3
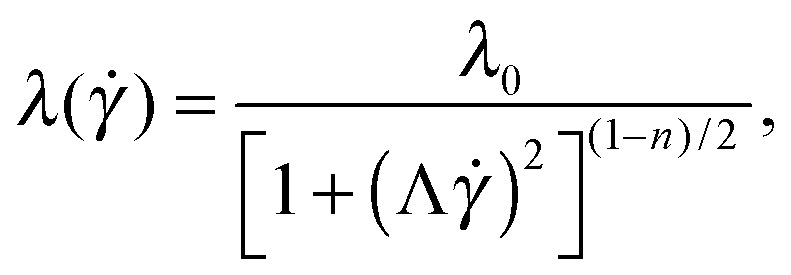

4 *N*_1_ = 2[*η*(*γ̇*) – *η*_s_]*λ*(*γ̇*)*γ̇*^2^,

where *η*_p,0_ and *λ*_0_ are the values of the polymer viscosity and relaxation time recovered in the limit of small shear rates. The parameter Λ sets the typical time scale for which non-linearities in the fluid response start to be noticeable, and the power-law exponent *n* sets the degree of shear thinning. Note that the model used here considers both the same Λ and *n* parameters for the shear viscosity and relaxation time (eqn (2) and (3)). Accordingly, the shear modulus, which relates polymer viscosity and relaxation time *G* = (*η*(*γ̇*) – *η*_s_)/*λ*(*γ̇*), is constant in the model used.

Shear-rate dependence of the shear relaxation time of polymeric solutions has been poorly reported in the literature.^35,36^ Typically, the relaxation time of complex fluids has been measured solely within the linear viscoelastic regime, for which the relaxation time is constant by definition.^10,28^ The theoretical work of Fielding and Olmsted^37^ uses a shear-rate dependence for the relaxation time of wormlike micellar solutions, but the amount of experimental data is limited for these systems.

We determine here the shear-rate dependence of the relaxation time following the White–Metzner model (eqn (4)) from the ratio of the first normal-stress difference *N*_1_ to the total shear stress *σ* = *η*(*γ̇*)*γ̇* as5
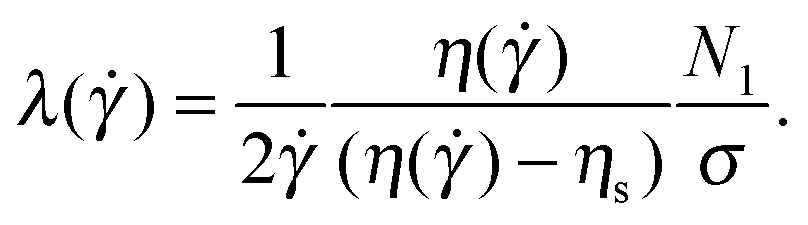
 Experimentally both *σ* and *N*_1_ can be directly measured with the rheometer, as well as the total shear viscosity *η*(*γ̇*) and the polymer viscosity contribution, *η*(*γ̇*) – *η*_s_, and thus the relaxation time can be computed. The success of this experimental methodology, however, relies on the quality of the measurement of *N*_1_, and thus the resolution of the normal force sensor of the (commercial) rheometers. This becomes particularly challenging at low shear rates and low polymeric concentrations. In order to improve the reliability of our data we performed these measurements using both the strain and stress-controlled rheometers with their corresponding geometries. To circumvent instrumental effects such as drift of the normal force, and to obtain reproducible data, we followed a specific shearing protocol described in more detail in the ESI.† The contribution of inertia to the normal force was also subtracted.^38^

**Fig. 2:**
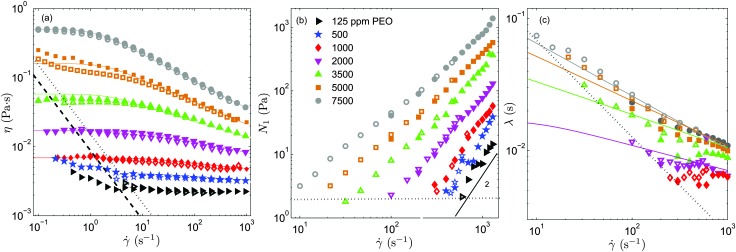
Shear viscosity (a), first normal-stress difference (b) and relaxation time (c) as a function of shear rate, measured at *T* = 23 °C, for different polymer concentrations. The measurements were performed using an ARES-G2 strain-controlled rheometer (empty symbols) and an Anton-Paar stress-controlled rheometer (filled symbols). Colour lines are fits to the experimental data obtained with the Anton-Paar rheometer, using the White–Metzner model. The dotted and dashed lines in panel (a) indicate the rheometer low-torque limit of both the ARES and Anton-Paar rheometers (the specifications of the manufacturer have been multiplied by 5), respectively. By measuring the *N*_1_ response for the Newtonian solvent (which should be identically zero) we determined experimentally the limit of low *N*_1_ that can be resolved by the rheometer (these data are obtained using the ARES-G2 rheometer) which is shown in panel (b) with a dotted horizontal line. A line of slope 2 is included in panel (b) as a guide to the eye. The resolution of the relaxation time obtained using our experimental methodology is also indicated in panel (c) using a dotted line.

In Fig. 2a and b we show the steady shear flow curves (shear viscosity and *N*_1_ *vs.* shear rate) for the various polymer concentrations measured at *T* = 23 °C. In the dilute regime (*c* = 125 and 500 ppm) the shear viscosity remains nearly constant for the entire range of shear rates measured (which largely covers the range of shear rates explored in the serpentine flow experiments) and *N*_1_ displays a quadratic dependence on the shear rate, as predicted by the Oldroyd-B model.

As the PEO concentration increases the total shear viscosity (which takes into account both polymeric and solvent contributions, *η* = *η*_p_ + *η*_s_) plateau (recovered for small shear rates) increases gradually. In addition, the fluid response becomes non linear. The degree of shear thinning for the shear viscosity becomes progressively more pronounced, and the slope of *N*_1_ as a function of shear rate in log–log coordinates becomes smaller than 2, and decreases progressively with the increase of polymer concentration.

In Fig. 2c we show the results obtained for the relaxation time using eqn (5). For *c* = 1000 ppm, we find that the relaxation time is nearly insensitive to the shear rate, *λ* ≃ 6 ms (within the experimentally accessible range of shear rates). This is the smallest relaxation time that we can resolve experimentally with our protocol with reasonable accuracy. For *c* < 1000 ppm experimental uncertainties in *N*_1_ and (*η*(*γ̇*) – *η*_s_) lead to considerable unphysical fluctuations of the relaxation time. Within the dilute regime the relaxation time is theoretically independent of polymer concentration.^22^ Therefore, we use for *c* = 125 and 500 ppm *λ* = 6 ms, the value obtained experimentally for *c* = 1000 ppm. Considering the important variability reported between different batches of nominally identical PEO,^39^ this value is in reasonable agreement with previous findings using similar PEO solutions.^14^

The coloured lines in Fig. 2 are fits of the White–Metzner model, which have been obtained by simultaneously fitting both *η*(*γ̇*) and *λ*(*γ̇*) for solutions with polymer concentration above c*. The model quantitatively captures the rheological response of PEO solutions in the semidilute regime, *c* ≥ 1000 ppm, for both shear viscosity and relaxation time. The rheological parameters obtained from the fits are shown in Table 1. As we have pointed out *c* = 1000 ppm is at the limit of the resolution of our experimental methodology. For this concentration it is not possible to measure shear thinning for the relaxation time, and we thus prefer not to fit the White–Metzner model in this case. For *c* = 1000 ppm, we include in Table 1 the measured value of *λ*_0_, and the exponent *n* that has been obtained using a power-law fit to the shear viscosity.

**Table 1:** Rheological parameters obtained for the polymeric solutions at different concentrations by fitting the White–Metzner model to the experimental data measured at temperature *T* = 23 °C

*c* (ppm)	*c*/c*	*η*_p,0_ (mPa s)	*λ*_0_ (s)	Λ (s)	*n* (–)
125	0.23	0.3	—	—	—
500	0.91	1.7	—	—	—
1000	1.83	4.8	0.006	—	0.88
2000	3.66	15	0.017	0.10	0.81
3500	6.40	56	0.05	0.50	0.75
5000	9.14	158	0.09	0.50	0.66
7500	13.71	498	0.15	0.80	0.60

The zero shear rate relaxation time *λ*_0_ increases with polymer concentration. Besides, for *c* > 1000 ppm the relaxation time displays a non-negligible shear-thinning behaviour (captured by the *n* exponent in the White–Metzner model). This shear-thinning behaviour becomes more significant as polymer concentration is increased. Since the degree of shear thinning predicted by the White–Metzner model for the first normal stress difference is more pronounced than the shear thinning for the shear stress, this model predicts a shear-thinning behaviour also for the relaxation time (determined *via*eqn (5)), as observed in our measurements.

**Fig. 3:**
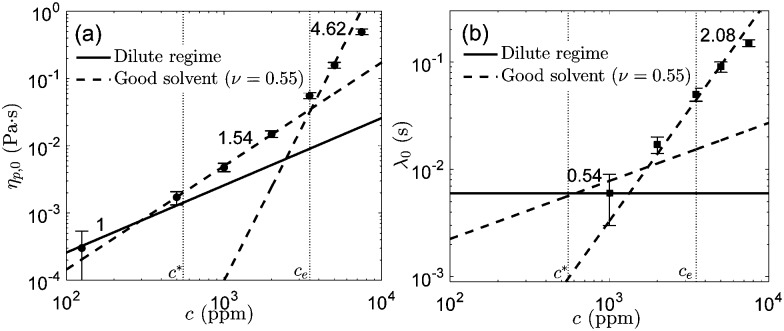
Polymer viscosity (a) and relaxation time (b) in the limit of the zero shear rate, obtained from the White–Metzner fit, as a function of polymer concentration. Continuous lines are fits to the data in the dilute regime. Dashed lines are the fits in the semi-dilute unentangled and semi-dilute entangled regimes obtained for a good solvent with *ν* = 0.55. The scaling exponents have been included for clarity. Dotted vertical lines indicate the overlap (c*) and entanglement (*c*_e_) concentrations.

In Fig. 3a we summarize the results obtained for the polymer shear viscosity in the limit of small shear rates (*η*_p,0_), for the whole set of solutions. Tirtaatmadja *et al.* ^40^ showed that water and mixtures of water and glycerol are good solvents for PEO, and determined experimentally the solvent quality, *ν* = 0.55 (which lies between the *θ*-solvent, *ν* = 0.5, and good solvent limits, *ν* = 0.588^22^). According to Rubinstein and Colby^22^ three different scaling regimes occur. For good solvents, in the dilute regime (*c* < c*) the polymer viscosity increases linearly with polymer concentration. In the semi-dilute unentangled regime (c* < *c* < *c*_e_, where *c*_e_ is the entanglement concentration) the scaling is given by *η*_p,0_ ∼ *c* ^1/(3*ν*–1)^, which leads to *η*_p,0_ ∼ *c* ^1.54^ upon substituting for the solvent quality. In the semi-dilute entangled regime (*c* > *c*_e_) *η*_p,0_ ∼ *c* ^3/(3*ν*–1)^, and thus *η*_p,0_ ∼ *c* ^4.62^. We performed fits to the experimental data using the prescribed scaling exponents for a good solvent with *ν* = 0.55 (included in Fig. 3a), and identified three different concentration regimes. Although we are conscious that the validity of these fits is questionable (because we have only a few experimental points), the fits show that our experimental data follow approximately the predicted trends. At low concentrations (*c* = 125 and 500 ppm) we recover the linear scaling corresponding to the dilute regime; for intermediate polymer concentrations (1000 and 2000 ppm) the viscosity increases with a power-law exponent close to 1.54, and for the largest concentrations (*c* = 3500, 5000 and 7500 ppm) the slope increases further, suggesting that for this polymer solutions *c*_e_ ≃ 3500 ppm. These scaling results are in relatively good agreement with experimental data reported by Ebagninin *et al.*,^27^ Arnolds *et al.*,^29^ Del Giudice *et al.*,^30^ for comparable PEO solutions in good solvents (although larger values of the entanglement concentration, *c*_e_ ≃ 4500^30^ and 6600 ppm,^27^ were estimated).

Alternatively, the scaling laws for the relaxation time in good solvents are predicted to follow *λ*_0_ ∼ *c* ^(2–3*ν*)/(3*ν*–1)^ in the semi-dilute unentangled regime (leading to *λ*_0_ ∼ *c* ^0.54^ for *ν* = 0.55) and *λ*_0_ ∼ *c* ^3(1–*ν*)/(3*ν*–1)^ in the semi-dilute entangled regime (*λ*_0_ ∼ *c* ^2.08^ for *ν* = 0.55).^22^ These are in relatively good agreement with the experimental work of van Zanten *et al.*,^23^ Zell *et al.*,^28^ Del Giudice *et al.* ^30^ for PEO solutions in good solvents. In Fig. 3b we show the dependence of the estimated zero-relaxation time *λ*_0_ as a function of polymer concentration, and include the corresponding fitting curves for completeness (although their relevance remains marginal because we have a few experimental points, and the uncertainty associated with the relaxation time estimate is significant).

### 3.2 Influence of polymer concentration on flow stability

**Fig. 4:**
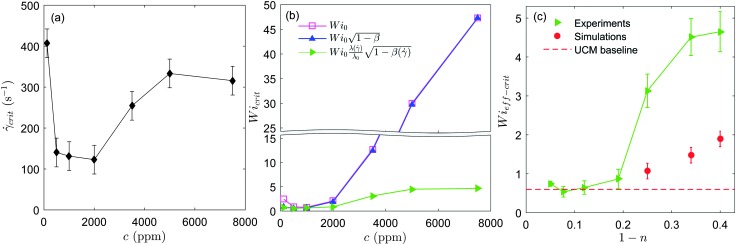
(a) Critical shear rate for the onset of elastic instabilities *vs.* polymer concentration measured in the microfluidic serpentine channels. (b) Critical Weissenberg number as a function of polymer concentration. The corrections for solvent viscosity and non-linear rheology are taken into account one at a time. (c) Critical effective Wi number *vs.* 1 – *n*, where *n* is the power-law exponent. Experimental results (green triangles) and 3D numerical simulations (red circles) obtained using the White–Metzner model are shown. The dashed red line corresponds to the situation of the absence of shear thinning obtained for the simulations using the UCM model.^14^

We tested the stability of the serpentine microflow for the set of PEO solutions at different polymer concentrations. We performed four independent runs for every solution. In Fig. 4a we plot the average critical shear rate, *γ̇*_crit_, for the onset of unstable time-dependent flows, as a function of polymer concentration. Our results show that the largest critical shear rate corresponds to the most dilute solution, with *c* = 125 ppm. Within an intermediate range of polymer concentrations (125 ppm ≤ *c* ≤ 2000 ppm), the critical shear rate progressively decreases as polymer concentration is increased. For higher polymer concentrations (*c* ≥ 3500 ppm) this trend is inverted and the critical shear rate increases with polymer concentration. Therefore, our observations reveal an initial destabilization flow stage (at moderate polymer concentrations) followed by a stabilization stage (at higher concentrations). Note that experiments performed using a larger radius of curvature, *R* = 1000 μm (not shown for conciseness), lead as expected to higher critical shear rates, but similar trends as a function of polymer concentration were observed.

Interestingly, we observed in the course of our rheological measurements in cone-plate shear flows comparable destabilizing trends as those reported in Fig. 4 for serpentine flows. In cone-plate flow measurements one can detect the departure from a viscometric flow, by an apparent shear-thickening behaviour in the measured flow curve (which can be attributed to the onset of elastic flow instabilities, if Re < 1). We observed that the critical shear rate for the onset of apparent shear thickening in cone-plate flow followed the same trends with polymer concentration as the ones reported for serpentine flows in Fig. 4 (data shown in the ESI†). Even though in the cone-plate flow the inertial component is not negligible, we suggest that the enhanced stabilizing effect may not be restricted to serpentine flows.

It is known that solvent viscosity has a stabilizing effect on the flow of viscoelastic solutions^15^ as was shown experimentally in the serpentine microflow of dilute polymeric solutions.^39^ By increasing the polymer concentration, the relative contribution of the solvent viscosity to the total shear viscosity (*η*_s_/*η*) decreases. For high polymer concentrations the effect of solvent viscosity becomes negligible (*η*_s_ ≪ *η*). In this concentration range, instead, the shear-thinning behaviour of polymer solutions becomes significant in the relevant range of shear rates.

To account for the influence of the solvent viscosity as well as the non-linear rheology of polymer solutions, we use the general expression of the instability criterion proposed by Pakdel and McKinley:^13^
6
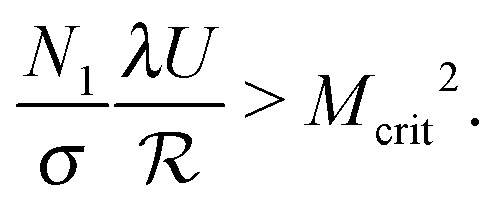
 The onset of elastic flow instabilities is given by a balance between the ratio of normal and shear stresses (*N*_1_/*σ*) and the ratio between a lengthscale for decay of flow perturbations (*l* = *λU*) and the streamline radius of curvature 
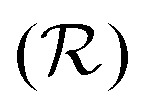
. *M*_crit_ is the instability threshold which depends on the flow geometry. In order to compare the relative strength of the flow for the different polymer solutions, it is useful to compute the dimensionless Weissenberg number, which is here defined as

7 Wi_0_ = *λ*_0_*γ̇*.

Note that we add the subscript 0 since we are using the zero shear rate relaxation time *λ*_0_ for this definition. For the UCM model (for which *η*_s_ ≡ 0), the Weissenberg number can be simply rewritten as 
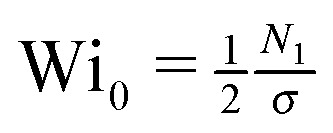
, leading to

8 Wi_0_ > *CM*_crit_,

for which we have expressed the mean velocity as *U* = *γ̇W*. The constant *C* is a geometric factor, function of the dimensionless average radius of curvature of the channel, 
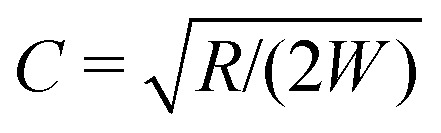
. For an Oldroyd-B fluid the Weissenberg number needs to be corrected for the solvent viscosity. This can be done, as suggested by McKinley *et al.* ^15^ by including the prefactor 
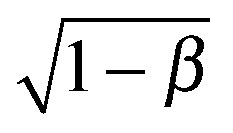
 (where *β* = *η*_s_/(*η*_s_ + *η*_p,0_) is the viscosity ratio). The instability criterion then reads,9
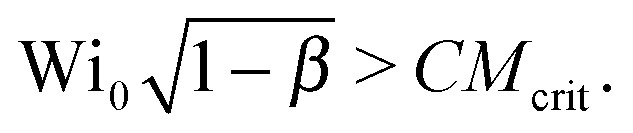
 Following McKinley *et al.* ^15^ a further step can be undertaken in order to account for the non-linear rheology of polymer solutions. This leads to a general expression of the instability criterion,10
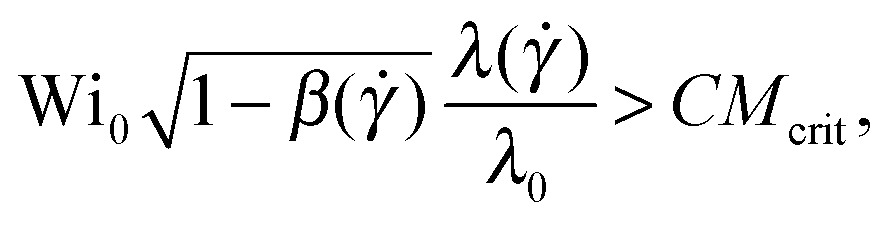
where the dependence of the relaxation time, *λ*(*γ̇*) as well as the polymer shear viscosity, *β*(*γ̇*) = *η*_s_/(*η*_s_ + *η*_p_(*γ̇*)), has been taken into account. We can define an effective dimensionless Weissenberg number equal to the left-hand side of eqn (10), as 
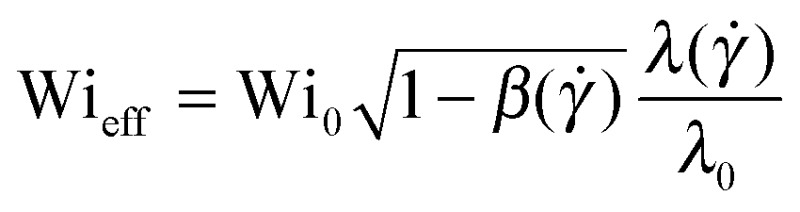
.

In Fig. 4b, we first plot Wi_0_ obtained for the onset of elastic instabilities, as a function of PEO concentration (squares). To test the influence of solvent viscosity on flow stability we then plot 
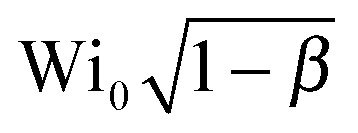
 (upper triangles). In the dilute regime (*c* = 125 and 500 ppm), including the solvent viscosity correction rescales *γ̇*_crit_ into a dimensionless instability threshold nearly independent of the polymer concentration. The slight differences observed between these results and previous results using similar PEO solutions^14^ can be attributed to the fact that the Zimm relaxation was used in Zilz *et al.* ^14^ as an estimate for the shear relaxation time.

We now take shear thinning into account by evaluating Wi_eff_ (side triangles). The effective Weissenberg number is independent of polymer concentration up to *c* = 2000 ppm. This indicates that Wi_eff_ correctly captures the effect of mild shear thinning on the instability threshold. But for larger polymer concentrations (*c* ≥ 3500 ppm), for which shear thinning becomes more important, the critical Weissenberg number displays a significant increase with concentration that persists, even after correcting for the shear-rate dependent rheology. This result suggests that the general Pakdel–McKinley criterion is not sufficient to explain the stabilization effect that we observe for shear-thinning polymeric fluids.

To investigate the stabilizing effect of shear thinning observed at high concentrations we compare our experimental results to 3D numerical simulations. This is shown in Fig. 4c. The critical Wi_eff_ number is plotted as a function of the shear-thinning exponent, 1 – *n*. The simulations were computed for the same geometrical conditions as used experimentally using a finite-volume method described in detail in Afonso *et al.* ^41^ To highlight the effect of shear thinning on the instability threshold, we have included a dashed red line corresponding to the numerical value of the critical Wi_eff_ number obtained by Zilz *et al.* ^14^ in the absence of shear thinning, using the upper-convected Maxwell model (UCM).

We observed previously in Zilz *et al.* ^14^ that experiments and simulations were in very good agreement as far as scaling laws were concerned, but slight quantitative differences were observed in the numerical prefactors of Wi_eff–crit_. This difference was attributed to the difficulty of determining precisely the relaxation times for such dilute polymer solutions and also to the limitations of the constitutive equations used. This also holds for experimental values obtained in the non-shear-thinning regime of the present study.

Our experimental results in Fig. 4c show that a clear departure from the non-shear-thinning scenario takes place for 1 – *n* > 0.2. Numerical simulations also show that the instability threshold for shear-thinning fluids is clearly higher than the threshold obtained for fluids with a constant viscosity (depicted by the dashed line in Fig. 4c). The instability threshold increases progressively with the degree of shear thinning. In both experiments and simulations shear thinning has a stabilizing effect on the serpentine flow, shifting the instability thresholds to progressively larger values, although the effect is less pronounced in the simulations. Overall, we observe a reasonably good qualitative agreement between experiments and simulations.

## 4 Discussion

We discuss in the following the possible influence of additional flow features (inertia, secondary flows, and the existence of a second normal-stress difference) on the microfluidic flow of polymer solutions. We will show that even if they can marginally contribute to the delay of the instability onset, none of them seems to be responsible for the significant flow stabilization that we report in this work.

In order to evaluate the role of fluid elasticity compared to flow inertia we compute the elasticity number, El = Wi_eff_/Re. At the onset of flow instabilities 0.5 < El < 87. Only for the most dilute solution is the elasticity number smaller than one, and inertia could thus have a non-negligible role. We performed experiments with the Newtonian solvent (with no elastic contribution) at comparable flow rates and verified that the flow remained stable. Therefore, the experiments were performed in a regime where inertia was negligible, and consequently inertial contributions are not at the origin of flow instabilities reported here.

Poole *et al.* ^42^ showed numerically that in serpentine flows of viscoelastic fluids secondary flows develop, even in the inertialess regime, due to the curvature of the geometry and the streamwise first normal-stress difference (which can be interpreted as the viscoelastic equivalent of Dean vortices, for Newtonian fluids). These results have recently been supported by experimental evidence in the flow of polymer solutions.^32,43^ The strength of secondary flows increases linearly with the applied flow rate. Note that for dilute polymer solutions^14^ secondary flows can be disregarded in the stability analysis, since at the critical flow rates for the onset of elastic flow instabilities these are still very weak. However, we have shown here that for shear-thinning fluids the instability onset shifts towards higher applied flow rates. At these largest flow rates secondary flows may be relatively strong, and it remains unknown whether they have an impact on flow stability (for example by advecting flow perturbations towards regions with a smaller flow curvature). Nevertheless, experiments performed by Larson *et al.* ^16^ in Taylor–Couette flows using shear-thinning polystyrene solutions also exhibited flow stabilization. Note that in the laminar large aspect ratio Taylor–Couette flows no secondary flows develop along the transverse direction (nor in cone-plate flows). We can therefore infer that secondary flows in serpentine channels, even though they cannot be disregarded, cannot be seen as the only mechanism responsible for flow stabilization.

The existence of second normal-stress difference, *N*_2_, may also lead to a stabilizing effect of the flow (as predicted theoretically by Larson *et al.* ^16^). However, for polymeric solutions the magnitude of *N*_2_ is small compared to *N*_1_ (only beyond the crossover to the entanglement regime, *c* > *c*_e_, the contribution of *N*_2_ may become more important^10^). Doi and Edwards^44^ predicted that *N*_2_/*N*_1_ = –2/7 at low shear rates for well entangled polymers. We estimated the influence of *N*_2_ using the Giesekus constitutive equation and the estimate of Doi and Edwards on the instability criterion, following the correction suggested by McKinley *et al.* ^15^ However, by including the existence of *N*_2_ in the scaling criterion the onset of elastic instabilities is modified by less than 20%. Thus, we believe that the stabilization of the instability observed cannot be captured by a simple rescaling of the Pakdel–McKinley criterion (even when including shear thinning and *N*_2_) suggesting a possible new instability mechanism.

## 5 Conclusions

We report here the stabilizing effect of shear thinning on the onset of elastic instabilities in serpentine flows, observed both experimentally and numerically. The shear rheology of dilute and semi-dilute PEO polymer solutions is reasonably well captured by the White–Metzner constitutive equation. For the model used, the polymer shear viscosity and the relaxation time display the same degree of shear-thinning, which becomes progressively more important as polymer concentration is increased. Our experimental results obtained in a serpentine microchannel reveal that using the scaling arguments of McKinley *et al.*,^15^ taking the contribution of the solvent viscosity as well as the shear-thinning rheology into account, captures the instability onset for weak shear thinning (*n* ≥ 0.8). For stronger shear thinning (0.6 ≤ *n* ≤ 0.8), however, an increase of the critical Weissenberg number is observed, corresponding to a stabilization of the flow. While this trend is not captured by the scaling argument for the instability criterion, it is in qualitative agreement with the 3D numerical simulations using the White–Metzner constitutive equation. The role of non-negligible *N*_2_ and secondary flows may be at play for this flow stabilization, but at the present stage it is not possible to discern their precise influence. Numerical simulations using a more complex constitutive model (such as the Phan-Thien–Tanner or the Giesekus model^10^) that captures the existence of *N*_2_ would enable these scenarios to be explored. Currently, the exact mechanism leading to the flow stabilization is still missing, and we hope that the precise quantification of the stabilization as a function of the degree of shear thinning presented here will stimulate the development of such a framework.
