# Supplementary material for: The stabilizing effect of shear thinning on the onset of purely elastic instabilities in serpentine microflows
Source: Soft Matter. 2016 May 17;12(29):6167–75. doi: 10.1039/c6sm00326e (PMC5043144; doi:10.1039/c6sm00326e)
Supplement: Supplementary file 1 [file SM-012-C6SM00326E-s001.pdf]

Electronic Supplementary Material (ESI) for Soft Matter.
This journal is © The Royal Society of Chemistry 2016
CREATED USING THE RSC LaTeX PCCP ARTICLE TEMPLATE – SEE www.rsc.org/electronicfiles FOR DETAILS

Supplementary Information: Stabilizing effect of shear thinning on the onset of purely elastic instabilities in serpentine microflows

Laura Casanellas, Manuel A. Alves, Robert J. Poole, Sandra Lerouge and Anke Lindner

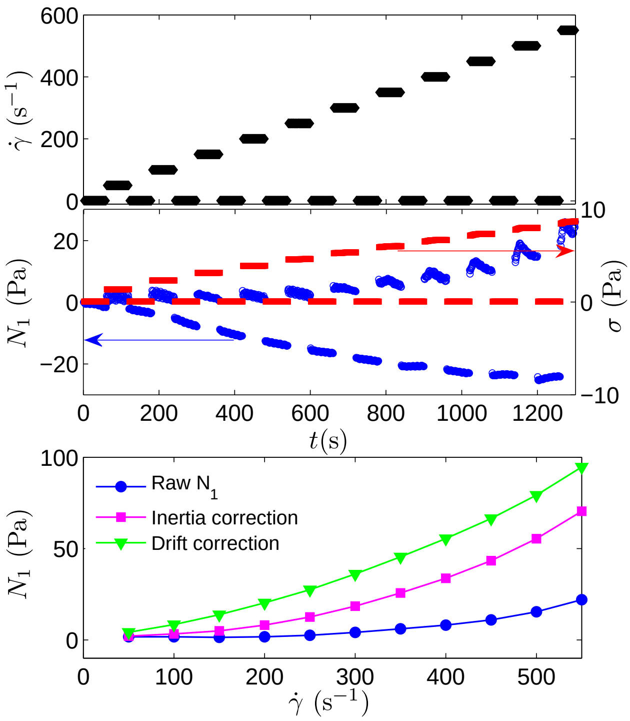

Fig. S 1 The experimental protocol we designed consisted of imposing a flow sweep test in which the solution was sheared at a small shear rate of reference, after every shearing step (top panel). This allowed us to reset the normal force in each step. The data obtained for the 3500 ppm PEO solution is shown in the central panel (the shear stress and N_1 are displayed in red squares and blue circles, respectively). The inertial contribution to the normal force was also subtracted. In the bottom panel we show the data corrected only for the inertial contribution (squares) as well as the data corrected for both inertial contribution and instrumental drift (lower-triangles).

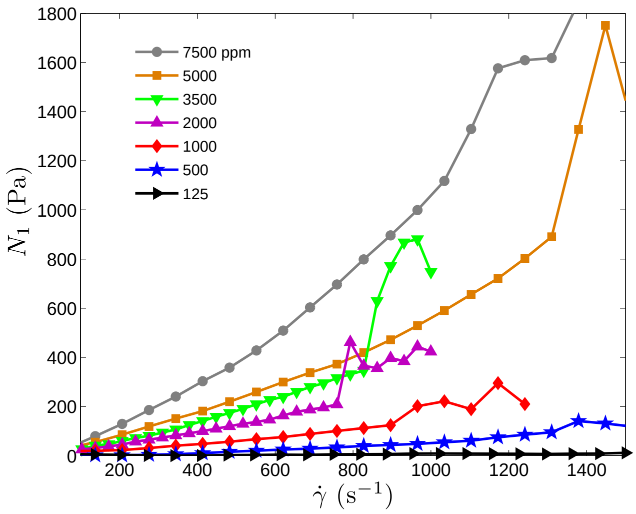

Fig. S 2 First normal stress difference as a function of shear rate. Measurements obtained using a stress-controlled rheometer (Physica MCR 501 from Anton Paar) and a cone plate geometry (with a diameter of 60 mm and 1° titanium cone). The onset of flow instabilities follows the same trends with polymer concentration as observed in the serpentine microchannel (Fig. 1c in the article). The temperature for these measurements was set at $T = 10^\circ$ C (in contrast to the rheological measurements reported in the manuscript which were all performed at $T = 23^\circ$ C). We set this lower temperature in order to increase the normal stress differences (N_1) of the polymer solutions and thus shift the onset of elastic instabilities towards lower shear rates, experimentally accessible with the rheometer.
